# Endoscopic ultrasound-guided selective inflow vessel devascularization: a novel approach in treatment of large gastric varices

**DOI:** 10.1055/a-2697-2542

**Published:** 2025-09-18

**Authors:** Bingru Qin, Aimin Li, Side Liu, Kangmin Zhuang

**Affiliations:** 1 198153 Department of Gastroenterology, Southern Medical University Nanfang Hospital, Guangzhou, China

Endoscopic ultrasound (EUS)-guided therapy has emerged as a pivotal advancement in interventional endoscopy for portal hypertension management. While conventional EUS-guided coil-assisted cyanoacrylate injection targeting variceal clusters has demonstrated efficacy in reducing glue-related complications, optimal material utilization remains challenging in cases of extensive varices 1 2 3 . We present an innovative approach utilizing EUS-guided selective inflow vessel devascularization (EUS-SIVD) to achieve precise hemodynamic control.

A 64-year-old woman presented with a 35 × 30-mm variceal cluster on the posterior wall of the gastric fundus near the dome ( Fig. 1 **a** ), type GOV2. We opted for EUS-guided precision therapy for gastric varices ( Video 1 ) after obtaining the patient’s consent.

**Fig. 1:**
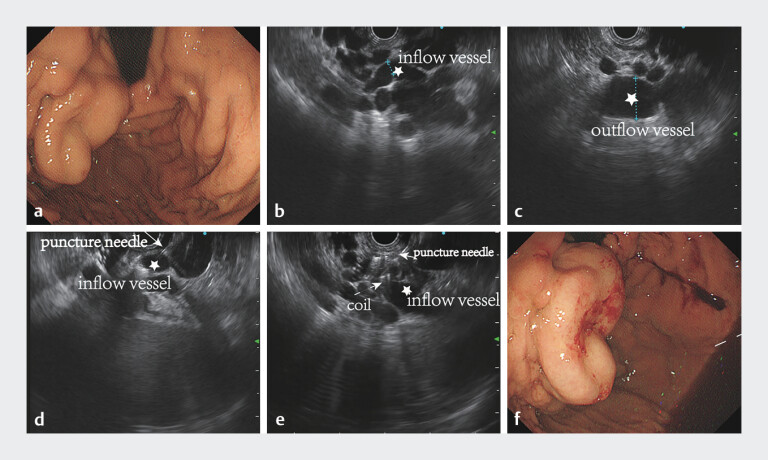
Endoscopy and endosonography images. **a** Endoscopy showed a large variceal cluster (35 × 30 mm) on the posterior wall of the gastric fundus near the dome, type GOV2. **b** Inflow vessel (diameter 6 mm). **c** Outflow vessel (diameter 19 mm). **d,e** Puncturing the inflow vessels and injecting the coils with cyanoacrylate glue. **f** Variceal cluster after the treatment.

Endoscopic ultrasound-guided selective inflow vessel devascularization for treatment of extensive varices.Video 1

Given the large volume of varicose vein masses and the existence of gastric-kidney shunt in the patient, direct targeting of the varicose vein masses may increase the dosage of tissue glue and raise the risk of ectopic embolization. Using EUS, we precisely traced two extramural inflow vessels leading into the intramural segment ( Fig. 1 **b, c** ) and punctured them. After placing three coils (MWCE 35–14–20 and 35–20–20; Cook Medical, Bloomington, Indiana, USA), 0.5 mL of cyanoacrylate glue with 0.5 mL of 50% dextrose was immediately injected ( Fig. 1 **d, e** ). Color Doppler flow imaging showed that most of the blood flow to the gastric fundal variceal cluster had been occluded just 2 minutes after the procedure ( Fig. 2 ). White-light endoscopy confirmed there was no significant bleeding at the puncture sites ( Fig. 1 **f** ). The total procedure duration was 20 minutes.

**Fig. 2:**
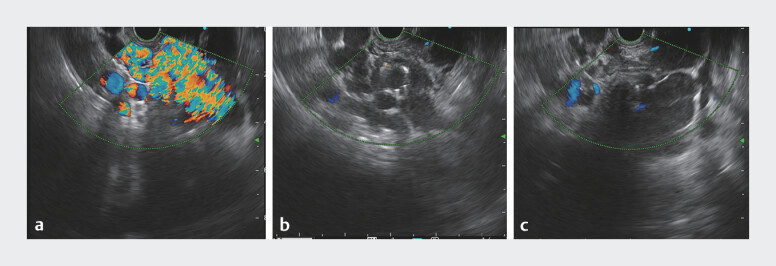
Color Doppler flow imaging. **a** Blood flow signal prior to treatment. **b** Blood flow signal after treatment. **c** The blood supply to the cluster was completely occluded.

Compared with traditional techniques, this novel method of EUS-SIVD precisely occludes the inflow vessels with EUS-guided treatment, leading to the use of significantly less cyanoacrylate glue and coils. This approach may decrease the likelihood of ectopic embolization and the risk of recurrence of gastric fundal varices, thus improving patient outcomes.

Endoscopy_UCTN_Code_TTT_1AS_2AB

## Notes

Endoscopy E-Videos https://eref.thieme.de/e-videos
*E-Videos* is an open access online section of the journal *Endoscopy* , reporting on interesting cases and new techniques in gastroenterological endoscopy. All papers include a high-quality video and are published with a Creative Commons CC-BY license. Endoscopy E-Videos qualify for HINARI discounts and waivers and eligibility is automatically checked during the submission process. We grant 100% waivers to articles whose corresponding authors are based in Group A countries and 50% waivers to those who are based in Group B countries as classified by Research4Life (see: https://www.research4life.org/access/eligibility/ ). This section has its own submission website at https://mc.manuscriptcentral.com/e-videos .
